# SLAMF7 as a Promising Immunotherapeutic Target in Multiple Myeloma Treatments

**DOI:** 10.3390/curroncol30090573

**Published:** 2023-08-27

**Authors:** Emily Chu, Jian Wu, Stacey S. Kang, Yubin Kang

**Affiliations:** 1 Division of Hematologic Malignancies and Cellular Therapy, Department of Medicine, Duke University Medical Center, Durham, NC 27710, USA; emily.chu@duke.edu (E.C.); jw731@duke.edu (J.W.); 2 Trinity College of Arts and Sciences, Duke University, Durham, NC 27708, USA; 3 College of Arts and Sciences, Washington University in St. Louis, St. Louis, MO 63130, USA; k.stacey@wustl.edu

**Keywords:** multiple myeloma, SLAMF7, antibody-based immunotherapy, elotuzumab, CAR-T

## Abstract

Multiple myeloma (MM) is a common hematological malignancy that has fostered several new therapeutic approaches to combat newly diagnosed or relapsed MM. While the field has advanced over the past 2 decades, the majority of patients will develop resistance to these treatments, causing the need for new therapeutic targets. SLAMF7 is an attractive therapeutic target in multiple myeloma, and a monoclonal antibody that targets SLAMF7 has shown consistent beneficial outcomes in clinical trials to date. In this review, we will focus on the structure and regulation of SLAMF7 and its mechanism of action. The most recent clinical trials will be reviewed to further understand the clinical implications and improve the prognosis of MM. Furthermore, the efficacy of anti-SLAMF7 monoclonal antibodies combined with standard therapies and possible resistance mechanisms will be discussed. This review aimed to provide a detailed summary of the role of SLAMF7 in the pathogenesis of patients with MM and the rationale for further investigation into SLAMF7-mediated molecular pathways associated with MM development.

## 1. Introduction

MM currently makes up 10% and is the second-highest of all hematological malignancies in the United States [1]. Every year, 32,000 patients are diagnosed with multiple myeloma, and 13,000 die from the disease; the prevalence increases with age [1]. MM characteristics include abnormal clonal plasma cells in the bone marrow. These abnormal clonal plasma cells are associated with monoclonal immunoglobin proteins or immunoglobin-free light chains in the serum or urine [2]. MM can present as plasmacytosis, paraprotein production, anemia, bone lesions, hypercalcemia, and renal damage. The pathophysiological characteristics include the suppression of antibody-mediated immunity, increased interleukin-6 levels, irregularities of the bone marrow microenvironment, and elevated osteoclastic activity [2]. Untreated, multiple myeloma can lead to excess production of monoclonal proteins or serum-free light chains and specific end-organ damage [3]. Novel therapies such as stem cell transplants, immunomodulatory agents, and proteasome inhibitors have been developed to treat MM. Furthermore, immunotherapy utilizing monoclonal or bispecific antibodies, antibody–drug conjugates, and CAR-T adoptive cell therapy has furthered the frontier of treatment research for MM. Despite these new treatments, many patients will develop resistance to these treatments, making them futile against MM.

Signaling lymphocyte activation molecule (SLAM) family receptors regulate the immune system through expression on hematopoietic cells. There are six SLAM family members: SLAM, CD229, 2B4, CD84, NTB-A, and CS1 [4]. The composition of SLAM molecules includes two or four immunoglobin (Ig)-like extracellular domains and immunoreceptor tyrosine-based switch motif (ITSM) intracellular domains. These molecules recruit small adaptor proteins: small adaptor cytosolic protein (SAP) or Ewing’s sarcoma-associated transcript 2 (EAT-2) [4]. As self-ligands, all of the SLAM family receptors, except for 2B4, have homotypic cell–cell interactions.

SLAMF7, also known as CS1/CRACC/CD319, promotes myeloma cell proliferation and growth due to its upregulation and high expression in MM cells. Immune cells such as B cells, T cells, dendritic cells, NK cells, and monocytes also express SLAMF7. However, the levels of SLAMF7 expression vary; it has high expression in plasma cells but low expression on inactive macrophages.

Some novel therapies that target SLAMF7 in MM cells include elotuzumab (a humanized IgG1 kappa monoclonal antibody) and chimeric antigen receptor-T cells (CAR-T). These have been shown to improve the prognosis of MM patients. Multiple clinical trials have been performed testing different combinations of elotuzumab and other immunomodulatory agents. However, MM is still considered an incurable disease, with many patients developing resistance to these treatments. A better understanding of the SLAMF7 signaling pathway and its clinical implications is important in the development of SLAMF7-targeted therapies for improved outcomes.

## 2. SLAMF7 Characteristics (Structure, Gene Expression, Function, and Polymorphism)

### 2.1. Structure

**Figure 1:**
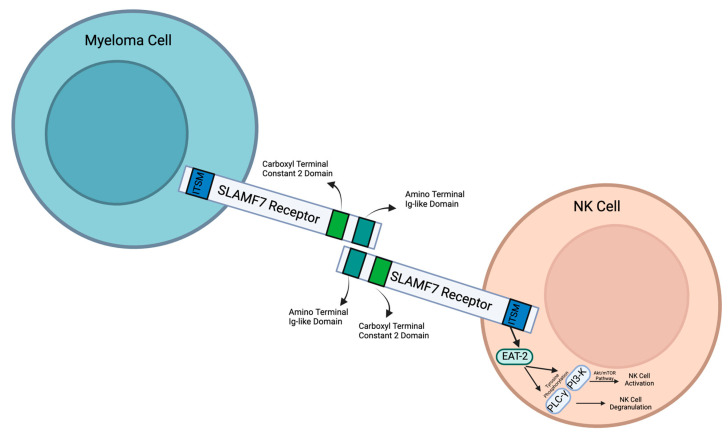
Structure, interaction, and downstream events of signaling lymphocytic activation molecule F7 (SLAMF7) in myeloma cells and natural killer (NK) cells. Created with Biorender.com.

As a member of the SLAM family of receptors, SLAMF7 possesses similar qualities. It is involved in cytotoxicity, humoral immunity, autoimmunity, cell survival, cell adhesions, and lymphocyte development [5]. SLAMF7 is conserved in chimpanzees, Rhesus monkeys, dogs, cows, mice, and rats, and 185 other organisms have orthologs with the SLAMF7 human gene. SLAMF7 is a 66 kDa type 1 transmembrane protein that is expressed on myeloma cells and immune cells [6,7]. There are three different domains: extracellular, transmembrane, and cytoplasmic (Figure 1).

The size of the SLAMF7 extracellular domain is 24 kDa [8]. It contains two immunoglobulin (Ig) superfamily domains: an amino terminal Ig variable domain and a carboxy terminal constant 2 domain (IgC2) [6]. These both have N-[9]glycosylation sites. There are 7 glycosylation consensus motifs: N56, N98, N142, N148, N172, N176, and N204. The most conserved glycosylation sites are N98, N142, and N148, and the most highly expressed glycosylation sites are N56, N98, and N148. It is suggested that N-glycosylation plays a role in cancer cell proliferation [9]. This amino terminal domain (IgC2) can bind with SLAMF7 extracellular domains through self-ligation. This then activates downstream signaling pathways.

SLAMF7 has a transmembrane domain that anchors the SLAMF7 receptor to the cell surface. In the cytoplasmic domain, there are four tyrosine residues. TVY_304_STV is inside an ITSM with a consensus sequence T-V/I-Y-x-x-V/I. TEY_284_DTI is inside in a similar ITSM-like sequence [6]. These sequences can signal downstream molecules and activate different intracellular pathways. For example, they control interactions with the SH2 domains of SH2D1A and SH2D1B [6]. Within cytoplasmic domains, there are two different subcategories: long cytoplasmic domain (SLAMF7-L) and short cytoplasmic domain (SLAMF7-S). Typically, the ITSM-like sequence contains SLAMF7-L. However, an mRNA splice variant encodes the SLAMF7-S receptor [6]. SLAMF7-S lacks two of the ITSM-like sequences and, as a result, does not interact with EAT-2. Alternatively, SLAMF7-S contains an alternative ITSM-like sequence (SKYGLL) due to a frame shift caused by the elimination of exon 5 [10]. NK cells usually express SLAMF7-L. SLAMF7-L is the activating isoform. SLAMF7-S is not involved in NK cell intracellular calcium flux or cytotoxicity and is not associated with SAP. More research must be done to discover the purpose of SLAMF7-S.

### 2.2. Gene

In humans, the SLAMF7 gene is located on chromosome 1 at locus 1q23-24 [11]. This gene comprises 12 kilobases and contains 7 exons. The first exon translates to the 5′ untranslated and leader sequence. The second exon encompasses the V domain, and the third exon codes for the C2 domain. The fourth exon translates into the transmembrane domain that anchors the receptor to the cell. The fifth through seventh exons encode the cytoplasmic domain [10]. This gene is regulated by several transcription factors that activate immune cells. It can also be epigenetically modified through DNA methylation and histone modifications, which can thus further affect its expression.

B lymphocyte-induced maturation protein, also known as positive regulatory domain zinc finger protein 1 (PRDM1) is a transcription factor of SLAMF7 [12]. BLIMP-1 binds at the −750 to −746 GAAAG sequence of the promotor of SLAMF7 [4]. BLIMP-1 likely serves a trans-activating function. It was also shown that SLAMF7 was expressed in cell lines that did not express Blimp-1. This suggests that Blimp-1 is not the only required transcriptional factor. It was reported that Yin Yang 1 (YY1) and a unique (AG)n = 36 DNA repeat element can bind to the mouse SLAMF7 promoter and regulate the transcription of mouse SLAMF7 [13]. While not required, Blimp-1 has been shown to increase transcription and expression of SLAMF7. It is significant to note that, while Blimp-1 is required for B and T cell differentiation, Blimp-1 is present in immature NK cells and increases in mature NK cells [12]. As a result, there are likely differences in the transcriptional regulation of CS1 in NK cells. However, more research is needed to further investigate the regulation of SLAMF7 transcription and expression.

### 2.3. Polymorphisms

Single-nucleotide polymorphisms (SNPs) are prevalent in the SLAMF7 promoter region, with one-point deletions and nine-point mutations, including the A > G mutation at −742 in the Blimp-1 binding motif [4,14]. The SLAMF3 rs509749 polymorphism is believed to correlate with malignant potential in MM [15]. It has also been found that SNPs significantly affect the differences in racial susceptibility to a wide range of diseases, including MM, and on the racial disparities in clinical outcomes in MM patients treated with high-dose melphalan and immunomodulatory agents [15,16,17,18,19,20,21,22,23,24,25]. Additional studies are warranted to determine whether polymorphisms in SLAMF7 affect the incidence and outcomes in different ethnic groups.

### 2.4. Function

NK cells are regulated through SLAMF7, which then plays a vital role in cancer recognition and antitumor responses. Through interactions between SLAMF7 and Ewing’s sarcoma-associated transcript 2 (EAT-2), NK cells are activated to recognize and target MM cells [26]. This interaction activates tyrosine phosphorylation of PLC-gamma and PI3K (Figure 1), which are two major regulators that activate NK cells. PLC-gamma activates NK cell degranulation, which releases cytotoxic granules to induce target cell death. The PI3K pathway activates the Akt/mTOR pathway. This further activates NK cells to produce cytokines, which stimulate degranulation. The cytokines that regulate NK cell activation are IL-2, IL-15, and IL-12. The SLAMF7 and EAT-2 interaction in NK cells enhances the lysis of harmful cells that express SLAMF7.

In T cells, the activation of SLAMF7 leads to the phosphorylation of STAT1 and STAT3 [27]. In contrast to NK cells, the phosphorylation of STAT1 and STAT3 in T cells does not strengthen the immune system towards tumor lysis. Instead, it causes the expression of multiple inhibitory receptors and transcription factors related to T cell exhaustion. Tumors utilize this strategy to evade the immune system and continue proliferating.

In B cells, SLAMF7 leads to the production of autocrine cytokines such as IL-14 [12,28]. As a result, it promotes B cell activity in contrast to inhibiting T cell activity.

Macrophages regulate the immune response against tumors and microbial pathogens. Macrophage-mediated phagocytosis is important in cancer control. It was demonstrated that SLAMF7 is indispensable in CD47 blockade-induced tumor cell phagocytosis, and this function of SLAMF7 relies on the interaction between SLAMF7 and integrin Mac-1 [29]. When these macrophages are no longer functional, they promote pathological inflammation. In super-activated macrophages, macrophages express SLAMF7 through IFN-gamma activation, which produces pro-inflammatory cytokines and chemokines defined as SLAMF7 super-activated macrophages (SLAMF7-SAMs) [30]. This then activates the nuclear factor kappa B (NF-κB) and mitogen-activated protein kinase (MAPK) pathways in addition to further autocrine amplification by TNF-α cytokine through an autocrine signaling loop [30]. As a result, SLAMF7 could be an important cue that drives pathology in acute and chronic inflammation. On the other hand, it was recently reported that SLAMF7 acts as a key suppressor of inflammation during sepsis [31]. SLAMF7 activates SHIP1, reduces K63-mediated ubiquitination of TNF receptor-associated factor 6 (TRAF6), attenuates MAPK/NF-κB signaling pathways, and downregulates macrophage proinflammatory cytokine production [31]. SLAMF7 protects mice from lethal sepsis [31].

In natural killer cells, SLAMF7 interacts with CRACC (CD2-like receptor) on the surfaces of target cells. This causes EAT-2, an SH2 domain-containing protein present in NK cells that is similar to SLAM-associated proteins (SAPs), to activate a downstream pathway [32]. Through an ITSM-like domain in the cytoplasm, EAT-2 binds to SLAMF7, which then promotes the tyrosine phosphorylation of PLC-γ and PI3-K [32]. These then regulate NK cell activation and degranulation, which allows the NK cells to have antitumoral and antiviral responses. If EAT-2 is not present in the cell, then this pathway is inhibited [32]. This impairs the anti-tumor immunity and then leads to the progression of MM [33].

In T cells, SLAMF7 promotes T cell exhaustion. As T cells undergo T cell receptor stimulation, they begin to lose their ability to produce cytokines. T cells that adopt an exhaustion phenotype are known to evade the immune response and allow tumors to proliferate. SLAMF7, in particular, inhibits receptors of CD8+ T cells [27,34]. SLAMF7 increases STAT1 and STAT3 phosphorylation [34]. STAT3 phosphorylation induces PD-1 checkpoint receptor expression. This inhibits effector T cells and promotes regulatory T cells, which express high levels of exhaustion markers [34]. The STAT1 mechanism is less understood, but it is likely that it increases open chromatin regions [34]. The role of SLAMF7 in dendritic cells remains to be investigated.

## 3. SLAMF7 in MM Pathogenesis and Disease Progression

SLAMF7 is highly expressed in myeloma cells and plays an important role in the pathogenesis of MM. In the bone marrow microenvironment, MM interacts with the extracellular matrix (ECM). This includes bone marrow stromal cells (BMSC), which can then activate MAPK, NOTCH, and Pi3K signaling pathways [35]. MM is able to escape immune system recognition through growth in the BM microenvironment [35,36].

The BM’s composition (myeloid-derived suppressor cells, tumor-associated M2 macrophages, N2 neutrophils, Tregs, Bregs, and plasmacytoid dendritic cells) promotes the proliferation of MM through the secretion of cytokines and growth factors (IL-6, IL-10, MIP-11 α/β, TGF β, stromal-derived factor 1(SDF-1), and a proliferation-induced ligand) [35,36]. These support MM cell growth and survival. Through a proliferation-induced ligand (APRIL), B-cell maturation antigens (BCMA) become overexpressed, which then activates the AKT, MAPK, and (NF)-kB signaling cascades. This allows MM tumors to proliferate. APRIL also increases the expression of cdc258B and ACK1. ACK1 regulates MM progression, inhibition of apoptosis, and drug resistance, which further allows MM tumors to grow [36,37]. Furthermore, APRIL inhibits IL-6 MM cells from apoptosis and uses cyclin D-dependent G1/S cell cycle progression to increase MM cell growth [37]. These tumors specifically have unique features, such as increased CD31 density and vascular endothelial growth [37].

In the majority of MM patients, SLAMF7 mRNA and protein are expressed in CD138+ tumor cells [26,38]. Using short interfering RNA that targets SLAMF7, it has been seen that MM cells no longer adhere to bone marrow stromal cells (BMSCs) [26]. This shows that it is likely that SLAMF7 mediates MM cell adhesion to BMSCs [38].

While there is no EAT-2 in myeloma cells, SLAMF7 uses other mechanisms to promote survival. SLAMF7 is important for myeloma cell interactions with bone marrow stromal cells. This can activate the ERK1/2, STAT3, and AKT pathways to promote survival [39]. However, more research must be done to understand these pathways.

Soluble forms of different immune-associated molecules were detected in different cancer patients. SLAMF7 has both a membrane-bound isoform and a soluble isoform. The level of surface expression of SLAMF7 did not correlate with MM disease progression. However, soluble SLAMF7 (sSLAMF7) reflects the disease progression of MM. The cleaving mechanism to produce soluble SLAMF7 is currently unknown. sSLAMF7 is found in the serum of 31% of MM patients, and no sSLAMF7 is found in healthy patients [26]. These sSLAMF7-positive patients had aggressive clinical characteristics and a shorter progression-free survival time than patients who did not have sSLAMF7 [26]. Significantly more sSLAMF7-positive patients were in the Revised International Staging System III (R-ISS). As a result, sSLAMF7 could be a potential biomarker.

Similar to SLAMF7, sSLAMF7 can interact with surface SLAMF7 on MM cells. Immunofluorescent staining has shown that activating SHP-2 and ERK coincides with sSLAMF7 binding to MM cells. However, instead of activating pathways that kill MM, SHP-2 and ERK signaling pathways promote tumor growth. Through ERK kinase activation, SHP-2 allows tumors to proliferate [40]. SHP-2 and ERK were phosphorylated, and ERK underwent nuclear translocation in SLAMF7 cells [40]. As a result, sSLAMF7 interacts with SLAMF7 on MM cells, which then activates these two pathways that promote MM proliferation.

## 4. Targeting SLAMF7 for MM Treatment

There are two main treatments for SLAMF7 in MM: elotuzumab and CAR-T cell therapy. While these treatments have proven to be affective, many patients relapse or develop resistance.

### 4.1. Elotuzumab

Elotuzumab (Empliciti^®^) is a humanized immunoglobulin G kappa antibody [41]. It targets SLAMF7 and activates NK cells through NK cell-mediated antibody-dependent cellular cytotoxicity or directly activating NK cells [6,33,42]. The cytotoxicity of NK cells is directly increased with elotuzumab through some pathways. NK T-cells are activated by elotuzumab binding, which then accelerates the secretion of IL2 and TNFα [43]. This enhances granzyme B release, which increases the cytotoxicity of NK cells against MM cells. In addition, elotuzumab can induce selective lysis of tumor cells through antibody-dependent cell-mediated cytotoxicity (ADCC). Elotuzumab binds its Fab portion to SLAMF7 on MM cells and the Fc portion to FcgRIIIa/CD16 on NK cells [44]. This increases CD69 and CD69MFI expression, which then triggers NK cell-mediated cytolytic activity [45]. While elotuzumab shows promise, it isn’t as prevalent in monoclonal antibody therapies used to treat MM.

#### 4.1.1. sSLAMF7 as a Predictive Biomarker for Elotuzumab Therapy

In regard to the two different spliced isoforms of SLAMF7 found in humans, SLAMF7-S did not have increased cytotoxicity by NK cells when compared to SLAMF7-L [43]. This suggests that the cytotoxicity of elotuzumab has to do with the ITSM cytoplasmic signaling motif, which is not present in the SLAMF7-S isoform.

Elotuzumab prevents the adhesion of MM to bone marrow stem cells, thus releasing it from cell adhesion protection. When elotuzumab targets SLAMF7, it blocks the MM cell from adhering to the bone marrow stem cells, which then enhances the sensitivity of the cell to chemotherapy.

There is a soluble form of SLAMF7 that can be used as a predictive biomarker for elotuzumab therapy. The concentration of sSLAMF7 decreases after binding by elotuzumab [42]. There were 82 patients in the phase 2 clinical trial CA204-116 that were randomized into two groups: elotuzumab-lenalidomide-dexamethasone (Eld) and lenalidomide-dexamethasone (Ld). Serum was collected before and after the treatment, and sSLAMF7 levels were analyzed. It was shown that there was a decrease in sSLAMF7 after treatment with Eld and Ld [7]. This indicates that elotuzumab neutralizes sSLAMF7. This was then compared to the overall response rate (ORR). It was seen that there was an increase in the portion of patients achieving a very good partial response (VGPR) or better in sSLAMF7 high patients who received elotuzumab, but there was no change in the portion of patients achieving a VGPR or better in sSLAMF7 low patients [7]. These clinical findings show that sSLAMF7 could be a predictive biomarker for the efficacy of elotuzumab in MM patients.

#### 4.1.2. Elotuzumab in Combination with Immunomodulatory Agents and Dexamethasone

While elotuzumab is a promising treatment, it has limited single-agent activity, suggesting that it must be used in combination with other anti-myeloma agents (Table 1). In a phase 1 study, there were no objective responses when elotuzumab was used alone to treat RRMM [46]. This suggests that monotherapy yields limited results.

ELOQEUNT-1 is an open-label, multicenter, randomized phase 3 trial. This trial is composed of patients with newly diagnosed multiple myeloma. It compares elotuzumab with lenalidomide and low-dose dexamethasone (Eld) to lenalidomide/dexamethasone (Ld) [47]. In this trial, 748 patients were enrolled, and 742 patients received treatment. The primary outcome was progression-free survival (PFS). PFS is the time from randomization to the first documented tumor progression or death. The results showed that the Eld regimen PFS was 31.38 months compared to the Ld regimen PFS of 29.47 months [47]. The statistical analysis (HR 0.93, stratified log-rank *p* = 0.44) showed that Eld and Ld did not significantly improve PFS for newly diagnosed, transplant-ineligible MM patients.

ELOQUENT-2 is an open-label, multicenter, randomized phase 3 trial for patients with relapsed or refractory multiple myeloma (RRMM) [48]. This trial had 646 patients with RRMM enrolled and randomly assigned to a treatment: elotuzumab combined with lenalidomide/dexamethasone or lenalidomide/dexamethasone. The primary outcome was measured using PFS and the overall response rate (ORR). The results showed that the PFS for elotuzumab combined with lenalidomide/dexamethasone was a median of 19.35 months, while the PFS for lenalidomide/dexamethasone was a median of 14.85 months (HR 0.70, *p* < 0.001). The ORR results for the percentage of patients that had a partial response or better on elotuzumab combined with lenalidomide/dexamethasone were 78.5%, whereas for lenalidomide/dexamethasone, it was 65.5%. After statistical analysis, it was shown that these results were statistically significant (*p* < 0.001). This indicates that the addition of elotuzumab improved treatment response and progression-free survival.

In addition, there was an open-label, multicenter, randomized, phase 2 clinical trial (ELOQUENT-3 trial) that combined elotuzumab with pomalidomide-dexamethasone (EPd) in comparison to just pomalidomide-dexamethasone (Pd) in patients with RRMM who failed lenalidomide and a proteasome inhibitor [49]. There were 117 RRMM patients enrolled in this trial, and they were randomly assigned to Pd or EPd regimens. The primary outcome was measured using PFS. The PFS for the EPd regimen was a median of 10.25 months, and the PFS for the Pd regimen was a median of 4.70 months. This was statistically significant (HR 0.54, *p* = 0.008), indicating that the addition of elotuzumab significantly impacts the survival of RRMM patients. The ORR was higher in the EPd arm (53%) compared to the Pd arm (26%) [49]. In the final overall survival (OS) analysis, EPd treatment was associated with significantly improved OS (29.8 months) versus the Pd arm (17.4 months, *p* = 0.0217) [50].

A phase 2 clinical trial utilizing elotuzumab, lenalidomide, and dexamethasone (ERd) was conducted on transplant-eligible MM patients. These 52 patients were newly diagnosed with MM. The trial consisted of four 28-day cycles of ERd and then an autologous stem cell transplant (ASCT) [51]. This was then followed by another four 28-day cycles of ERd treatment. The results were measured using the induction feasibility rate (IFR), which was the percentage of patients that finished the first four 28-day cycles and started ASCT. The IFR was 68.8%. The secondary outcome was measured using the complete response rate (CRR = 49%), the overall response rate (ORR = 92.2%), and progression-free survival (PFS = 29.7 months) [51]. These results show that the ERd regiment followed by ASCT was well tolerated in patients.

#### 4.1.3. Elotuzumab in Combination with Proteasome Inhibitor and Dexamethasone

CA204-009 (NCT01478048) is a phase 2 open-label, randomized study. There were 152 patients enrolled in this trial. There were two treatments compared: elotuzumab combined with bortezomib and dexamethasone (EBd) or dexamethasone (Bd) in RRMM patients [52]. The primary outcome was measured using ORR and PFS. The ORR for EBd was 65%, whereas the ORR for Bd was 63%. These results were not statistically significant. However, the PFS for EBd was 9.9 months. The PFS for Bd was 6.8 months. This was seen to be statistically significant (HR 0.60, *p* = 0.0116) [52].

Another trial studied the efficacy of elotzumab with carfilzomib and dexamethasone. There were 15 patients with RRMM enrolled in the study. They had all received 1–3 prior lines of therapy [53]. Carfilzomib was given at 70 mg/m^2^ every week for 3 weeks, and then 1 week off after 20 mg/m^2^ on C1D1. The primary outcome was measured using ORR and PFS. The ORR was 87%, with 7 (47%) patients achieving a VGPR or better. The median PFS was 22 months [53].

Finally, a phase 2 minimal residual disease (MRD)-adaptive trial with a treatment composed of elotuzumab with carfilzomib, lenalidomide, and dexamethasone (Elo-KRd) was investigated. The patients were newly diagnosed with MM. The study did not have specific transplant eligibility criteria [54]. The primary endpoint of the study was stringer CR (sCR) rate and/or MRD negativity rate after 8 cycles of Elo-KRd. Forty-four patients were enrolled, 39 of whom were evaluable for response. The rate of sCR and/or MRD(−) was 58%. The estimated 2-year PFS was 87%, and the estimated 2-year OS was 89%. Serious adverse events were observed in 30 patients (68%), and one patient died from myocardial infarction [54].

#### 4.1.4. Elotuzumab in Combination with other Anti-Myeloma Agents

Elotuzumab in combination with belantamab mafodotin (NCT05002816) or selinexor backbone regimens (NCT02343042) is on clinical trial. It remains to be determined how to sequence or combine elotuzumab with CD38-targeted monoclonal antibodies, such as daratumumab and isatuximab.

### 4.2. CAR-T

Chimeric antigen receptors (CARs) are receptors with an antigen-recognition domain. These are combined with a T-cell activation domain to make a specific type of immunotherapy known as CAR-T therapy, in which a patient’s T-cells are genetically modified to express a chimeric antigen receptor. This chimeric antigen receptor will then specifically target cancer cells, forgoing the need for human leukocyte antigen presentation. When this genetically modified T-cell binds to cancer cells, a signaling cascade is initiated that stimulates the release of cytokines (TNF-α, IFN-γ, IL2, and IL6) to destroy the tumor cell through cytolysis [55].

Using CAR-T therapy, a SLAMF7 chimeric antigen receptor was developed to target MM cells. This has proven to be effective [56]. CAR-T cells trigger selective fratricide of SLAMF7+/high lymphocytes such as NK cells, CD4+ and CD8+ T cells, and B cells. These CAR-T cells preserve functional lymphocytes [55]. These CAR-T cells internalize the SLAMF7 protein, which lowers the amount of SLAMF7 (high). This process allows CD8+ and CD4+ cells to achieve a SLAMF7 (low) phenotype [55,57]. Furthermore, a CD8+ fratricide-resistant SLAMF7 CAR-T was created. This specific CAR-T therapy was seen to protect functional CD8+ cultures [8]. Since SLAMF7 is expressed in NK cells, this CAR-T therapy can lead to NK cell death and deficiencies. This can lead to severe infections in patients. As a result, there is a suicide gene in the SLAMF7 CAR-T. The suicide gene encodes a dimerization domain with a caspase-9 domain [8,58]. This allows the elimination of the SLAMF7 CAR-T cells before NK cells become depleted and significant cytotoxicity occurs [58].

There is a phase 1 clinical trial on the SLAMF7 CAR-T and its efficacy and safety in targeting MM. There were 13 patients enrolled in this trial. These patients all had MM and had received previous treatment to no effect. This trial consisted of giving increasing doses of anti-SLAMF7-CAR+ T cells: level 1 of 0.66 × 10^−6^ per kg of body weight; level 2 of 2.0 × 10^−6^ per kg of body weight; level 3 of 6.0 × 10^−6^ per kg of body weight; and level 4 of 12.0 × 10^−6^ per kg of body weight. The primary outcome was measured by the number of participants who had a specific grade of adverse effects. The results showed that three patients could only tolerate level 1, three patients at level 2, three patients at level 3, and one patient at level 4.

There is another phase 1 trial that is testing CAR-T therapy after chemotherapy for patients with relapsed or refractory MM. This trial is investigating the side effects and optimum dosage of CAR-T therapy. There are 30 participants. The primary outcome is the incidence of dose-limiting toxicity, opportunistic infections, and prolonged lymphopenia. This trial is currently ongoing.

There is an in-human clinical trial with SLAMF7 CAR-T cells called CARAMBA that uses a virus-free Sleeping Beauty transposon. SB is the first transposon to show transposition in vertebrae cells by combining favorable parts of the viral vector with DNA molecules. The CAR cassette targeting domain is derived from elotuzumab. There are two phases to this clinical trial: phase 1—dose escalation; and phase 2a—dose expansion. The results of this trial are still pending [59].

There is an “off-the-shelf” allogenic anti-SLAMF7-CAR-T cell called UCARTCS1 [60]. Using TALEN gene editing technology, a healthy allogenic T cell was edited to eliminate endogenous TCR and SLAMF7 expressions in order to reduce the risk of the graft reacting against the host (GVHD) and T-cell fratricide [60].

Finally, there is a novel CAR-T product under preclinical development. This is an anti-SLAMF7/BCMA combined CAR-T cell. This is intended to simultaneously increase tumor coverage and decrease antigen loss. Through the two ligand-binding domains for SLAMF7 and BCMA, tumor lysis activity should be accelerated in vivo.

### 4.3. Resistance Mechanism to SLAMF7 Antibody

While SLAMF7 has shown to be a promising target for immunotherapy in multiple myeloma, many patients develop resistance, which then leads to treatment failure. However, the mechanism of resistance to elotuzumab is still unknown due to no single agent activity.

**Figure 2:**
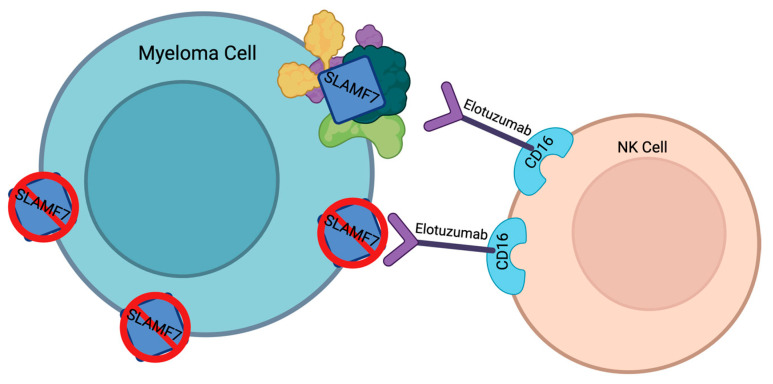
Resistance mechanism theories for elotuzumab. Created with Biorender.com.

One possible mechanism could be the downregulation of SLAMF7 expression in multiple myeloma cells (Figure 2) [61]. This decrease in target expression causes elotuzumab to be less efficient in treating MM. However, this mechanism has not been confirmed.

Another possible resistance mechanism could be the upregulation of other cell surface proteins or pathways (Figure 2) [61]. Thus, even though SLAMF7 signaling is lost, the multiple myeloma cells can continue to survive and proliferate. As a result, elotuzumab’s efficacy decreases.

## 5. Discussion

Multiple myeloma is an incurable disease that impacts a significant portion of the population. SLAMF7 is upregulated in MM and plays a role in the proliferation of cancer cells. SLAMF7 interacts with other proteins on NK cells to regulate tumor response. SLAMF7 can also interact with different proteins to control the regulation of T cells, B cells, and macrophages. While there has been increased interest in and research on this protein, the specific pathways of SLAMF7 need further investigation. The role of SLAMF7 in myeloma cell proliferation isn’t entirely clear yet. SLAMF7 likely promotes bone marrow adhesion, by interacting with the bone marrow microenvironment to hide from the body’s immune system, resulting in the proliferation of MM cells.

There have been many clinical trials of elotuzumab combined with other anti-myeloma therapeutics. Elotuzumab combined with pomalidomide has shown the most promising results in relapsed and refractory multiple myeloma. More research should be done to understand the mechanism behind this drug-inhibiting multiple myeloma. It is suspected that the mechanism involves the soluble form of SLAMF7, which can also be used as a biomarker since it is elevated in MM patients but not present in healthy patients. Identifying biomarkers to monitor treatment efficacy can improve treatment response. While sSLAMF7 shows promise as a biomarker of MM, more research has to be done to clarify the threshold between sSLAMF7 high/low patients.

While novel therapeutics such as elotuzumab and CAR-T have improved patient prognosis, MM is still incurable due to most patients developing resistance to the treatments. The resistance mechanisms still need to be fully investigated. Researching them could provide opportunities to combat this resistance or target new molecules in order to improve the prognosis of MM patients.

Further research can optimize the use of SLAMF7-targeted therapies and improve the outcomes of MM patients. BLIMP-1 has been shown to be a transcriptional factor for multiple myeloma. Targeting transcriptional factors could lead to a decrease in the expression of SLAMF7 in multiple myeloma cells. Therefore, new therapies targeting this or other downstream signaling pathways could improve outcomes for multiple myeloma patients.

Overall, while SLAMF7 shows a lot of promise in the treatment of MM, more research must be done to develop new and lasting treatments.

## Figures and Tables

**Table 1:** Summary of elotuzumab-based regimens in the treatment of multiple myeloma *.

Regimen	Trial	Patient Population	Arms	Results	Reference
Elotuzumab monotherapy	Phase 1, open label, dose escalation	*n* = 35; RRMM	Elo single agent	No objective responses	Zonder et al. [46].
**Elotuzumab + IMiD +Dex**					
ELOQUENT-1	Open label, randomized, phase 3	*n* = 748; NDMM, transplant ineligible	ELd vs. Led	PFS: 31.38 months (ELd) vs. 29.47 months (Ld) (HR 0.93, *p* = 0.44)	Dimopoulos, M.A. et al. [47].
ELOQUENT-2	Open label, randomized, phase 3	*n* = 646; RRMM	ELd vs. Ld	PFS: 19.35 months (ELd) vs. 14.85 months (Ld) (HR 0.70, *p* < 0.001)	Lonial, S. et al. [48].
ELOQUENT-3	Open label, randomized, phase 2	*n* = 117; RRMM	EPd vs. Pd	PFS: 10.25 months (EPd) vs. 4.70 months (Pd) (HR 0.54, *p* = 0.008)	Dimopoulos, M.A. et al. [49,50].
Elo + Lenalidomide +Dex	Open label, phase 2	*n* = 52; NDMM, transplant eligible	ELd	ORR: 92.2%; PFS: 29.7 months	Innovations, S.D. [51].
**Elotuzumab + PI + Dex**					
CA204-009: Elo-Bor-Dex vs. Bor-Dex	Open label, randomized, phase 2	*n* = 152; RRMM	EBd vs. Bd	ORR: 65% (EBd) vs. 63% (Bd); PFS: 9.9 months (EBd) vs. 6.8 months (HR 0.6, *p* = 0.0116)	Palumbo, A. et al. [52].
Elo-Carfilzomib-Dex	Phase 2	*n* = 15; RRMM	Elo-Kd	ORR: 87%; PFS: 22 months	Silvennoinen, R.H. et al. [53].
Elo-Carf-Len-Dex (Elo-KRd)	Phase 2, MRD-adaptive	*n* = 44; NDMM	Elo-KRd	sCR/MRD-: 58%; 2 yr PFS: 87%; 2 yr OS: 89%	Derman, B.A. et al. [54].

*: RRMM: relapsed or refractory multiple myeloma; NDMM: newly diagnosed multiple myeloma; IMiD: immunomodulatory agent; PI: proteasome inhibitor; HR: hazard ratio; ORR: overall response rate; PFS: progression-free survival; OS: overall survival; MRD: minimal residual disease.
